# Cross sectional analyses indicated that body fat distribution was not associated with performance-based and self-reported outcomes among ambulatory people with multiple sclerosis

**DOI:** 10.1016/j.msard.2026.107084

**Published:** 2026-02-17

**Authors:** Ariel Kidwell-Chandler, Brenda Jeng, Robert W. Motl

**Affiliations:** Department of Kinesiology and Nutrition, College of Applied Health Sciences, University of Illinois Chicago, Chicago, IL, USA

**Keywords:** Multiple sclerosis, Dual-energy x-ray absorptiometry, Body composition, Body fat distribution, Visceral adipose tissue

## Abstract

**Background:**

Obesity is cited as a relevant factor in multiple sclerosis (MS). To date, there is inconsistent evidence regarding the relationship between total body fat and frequently studied outcomes in MS. Body fat distribution, or the location of adipose tissue and depots within the body, may represent a biologically meaningful correlate of MS-related outcomes.

**Purpose:**

This exploratory study assessed whether dual-energy X-ray absorptiometry (DXA)-based body fat distribution metrics were associated with performance-based and self-reported outcomes of MS.

**Methods:**

Fifty-seven ambulatory adults with MS underwent DXA to quantify visceral and subcutaneous adipose tissue; android- and gynoid- % fat mass; and android/gynoid ratio. Outcomes included performance-based (Timed Up and Go, Timed 25-Foot Walk, Six-Minute Walk) and self-reported assessments (Hospital Anxiety and Depression Scale anxiety and depression [HADS-D] sub-scales), Multiple Sclerosis Impact Scale physical [MSIS Physical] and psychological domains, Fatigue Severity Scale, and the short form of the McGill Pain Questionnaire). Spearman correlation assessed bivariate and partial (controlling for sex and age) associations.

**Results:**

Correlations between body fat distribution metrics and performance-based and self-reported outcomes were generally small and not statistically significant. Gynoid % fat mass correlated with depressive symptoms (HADS-D: *r*_*s*_ = −.28, *p* = .033) and physical health-related quality of life (MSIS Physical: *r*_*s*_ = −.31, *p* = .018), but partial correlations were not statistically significant.

**Conclusion:**

Our results did not support an association between body fat distribution and frequently studied MS-related outcomes. This aligns with research suggesting a limited role of adiposity and MS-related outcomes.

## 1. Introduction

Multiple sclerosis (MS) is a chronic, progressive, immune-mediated disease of the central nervous system with a prevalence nearing one million adults in the United States (McGinley and Goldschmidt, 2021, Wallin et al., 2019, Number of people with MS | Atlas of MS 2025). There are conceptual models indicating that environmental, social, behavioral, and genetic factors are associated with MS onset, outcomes, and progression (McGinley and Goldschmidt, 2021, Nourbakhsh and Mowry, 2019). Obesity, defined as excessive body fat (i.e., adipose tissue), is one of the factors conceptually associated with the development of MS and its expression (Nourbakhsh and Mowry, 2019, Schreiner and Genes, 2021, Correale and Marrodan, 2022). Obesity is associated with pro-inflammatory mechanisms underlying cardiovascular and metabolic conditions, and those conditions seemingly impact MS (Marrie et al., 2023, Hruby and Hu, 2015). Obesity-related comorbidities (e.g., diabetes, hypertension, and hyperlipidemia) are rising faster in incidence among those with MS than the general population and may partially explain the variability of disease burden in MS (Marrie et al., 2023, Marrie et al., 2016).

To date, the association between dual-energy X-ray absorptiometry (DXA)-based total body fat and outcomes has been far from conclusive in MS. One cross-sectional study in MS reported that neither whole-body fat mass nor percent (%) body fat were significantly associated with frequently studied outcomes in MS, including anxiety or depressive symptoms, fatigue, or mobility (Pilutti and Motl, 2019). Other cross-sectional studies have further reported mixed or no associations between whole-body fat mass or % body fat and performance-based or self-reported MS outcomes (Cozart et al., 2023, Jeng and Motl, 2022, Silveira et al., 2020). This generally supports considering facets beyond *overall* body fatness and outcomes in MS.

An important nuance when examining the association between obesity and MS outcomes might involve the distribution of body fat. Body fat distribution involves the location of adipose tissue and depots in the human body, and the location of body fat may represent a biologically relevant dimension within the broader obesity-MS relationship. Visceral adipose tissue (VAT; adipose tissue surrounding intra-abdominal organs) is notably inflammatory and associated with cardiometabolic diseases in the general population (Ruiz-Castell et al., 2021, Shuster et al., 2012). The amount of VAT may be associated with poorer disease outcomes and faster disease progression in MS (Giannopapas et al., 2024). Subcutaneous adipose tissue (SAT; adipose tissue between skin and muscles) is a more metabolically favorable fat depot than VAT in the general population (Guerreiro et al., 2022, Porter et al., 2009). SAT may confer less associations with MS outcomes and progression.

Another important nuance regarding body fat distribution is body shape. This representation of body fat distribution involves adipose tissue in the central or ‘android’ region (i.e., commonly referred to as “apple shape”) compared with the gluteofemoral or ‘gynoid’ region (i.e., “pear shape”) (Karastergiou, 2019). The android location of adiposity has been associated with cardiometabolic disease, whereas gynoid adiposity has not, and this is perhaps associated with the central anatomical location of VAT (Guerreiro et al., 2022, Karastergiou, 2019). The gynoid distribution of adiposity, on the other hand, may further offer a biomechanical advantage for mobility outcomes compared with the android region in MS (Ahn et al., 2024).

There is limited available research on body fat distribution and its association with outcomes in MS. Some research has associated VAT with MS pathogenesis and disability, but the studies have not systematically examined the association between body fat distribution and frequently studied outcomes of MS (e.g., mobility, anxiety and depressive symptoms, health-related quality of life, fatigue, and pain) (McGinley and Goldschmidt, 2021, Fitzgerald et al., 2020, Livne--Margolin et al., 2021). An even larger limitation is that researchers have predominantly utilized anthropometric measures such as waist circumference and/or waist-to-hip ratio, rather than high fidelity measures like DXA when quantifying body fat distribution (Kidwell-Chandler et al., 2025).

This exploratory study examined the associations of DXA-based body fat distribution with performance-based and self-reported outcomes of MS using secondary data analysis from a published study on body composition and oxygen cost of walking (Jeng et al., 2023). The results may offer insights into how patterns of body fat distribution, including a systemically inflammatory adipose tissue (i.e., VAT), may be associated with outcomes of MS.

## 2. Materials and methods

### 2.1. Setting and participants

This exploratory study involved a secondary analysis of data from a previously published study examining body composition and oxygen cost of walking (Jeng et al., 2023). This study was conducted in the southeastern region of the United States. Participants with MS were recruited between September 2021 through February 2022 via word-of-mouth and flyers distributed amongst local healthcare practitioners (e.g., neurologists and physical therapists), support groups, and the community. Participants were recruited across a range of body mass index (BMI) categories (normal [18.5 – 24.9 kilograms (kg)/meters squared (m^2^)], overweight [25 – 29.9 kg/m^2^], and obese [≥30 kg/m^2^] status) for yielding variation in body composition when examining associations between body fat and outcomes in MS (Number of people with MS | Atlas of MS 2000). The inclusion criteria were: (1) diagnosis of MS; (2) no relapse or sudden change in MS symptoms within the last 30 days; (3) ambulatory with or without an assistive device; (4) age 18 years and older; and (5) not pregnant. To confirm eligibility, participants completed a self-report screening questionnaire that assessed all inclusion criteria.

**Fig. 1.**
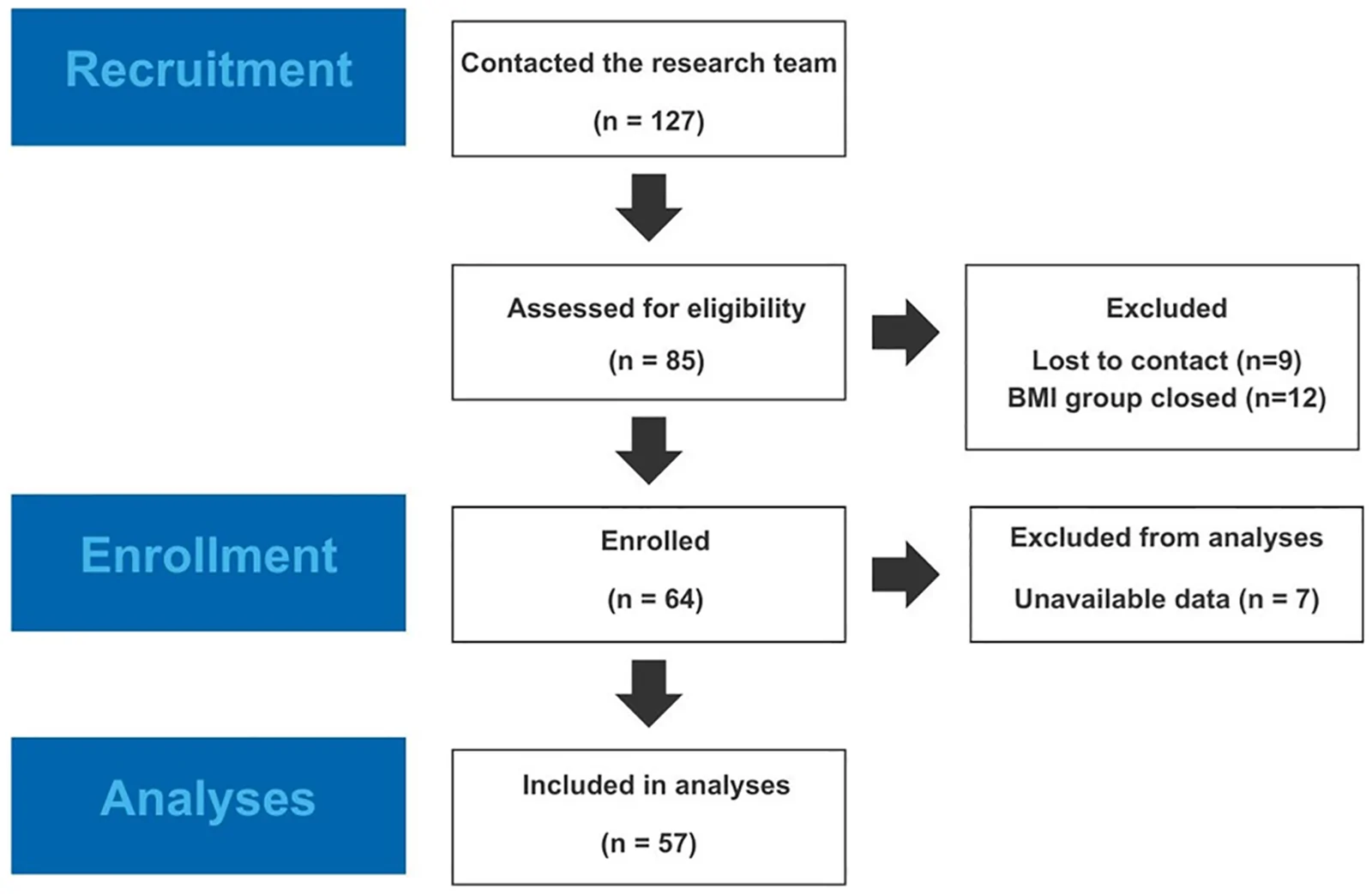
Participant CONSORT.

The sample size for the primary study was estimated through an *a priori* power analysis. The power analysis indicated that a sample of 64 participants would achieve 80 % power for detecting a one-tailed, moderately-sized bivariate correlation of .3 or larger (G*Power, Germany). The CONSORT in Fig. 1 indicates that there were 127 individuals who contacted the research team, and 85 were assessed for eligibility. Of the 85, 9 were lost to contact after screening, 12 were excluded due to the targeted BMI categories being closed for further recruitment, and 7 were excluded from analyses due to unavailable data relevant to the present study (e.g., no VAT data). The final sample for data analyses included 57 people with MS. Because this secondary analysis was exploratory and used two-tailed tests, the achieved power for detecting a correlation of .3 is lower than the original one-tailed target (i.e., approximately 65 % vs 80 %; G*Power, Germany).

### 2.2. Procedure

The procedure was approved by a University Institutional Review Board (University of Alabama at Birmingham), and all participants provided written informed consent. The data were collected during a single university-based laboratory visit. Participants provided demographic and clinical information and then completed performance-based and self-reported assessments and a DXA scan. Participants were remunerated for time and travel.

### 2.3. Measures

#### 2.3.1. Demographic, clinical, and morphological characteristics

Participants provided sex, age (years [yrs]), race, disease duration (yrs), duration of MS symptomology (yrs), MS type, and use of disease-modifying therapies (DMTs) on a lab-based questionnaire. Neurological status was based on the Expanded Disability Status Scale (EDSS) with the score generated by a Neurostatus-certified member of the research team (Kurtzke, 2015). To further characterize the sample, DXA-derived morphological characteristics, including BMI (kg/m^2^), % fat mass, % lean soft tissue mass, and bone mineral content (kg) were collected and summarized descriptively.

#### 2.3.2. DXA-based body fat distribution metrics

**Fig. 2.**
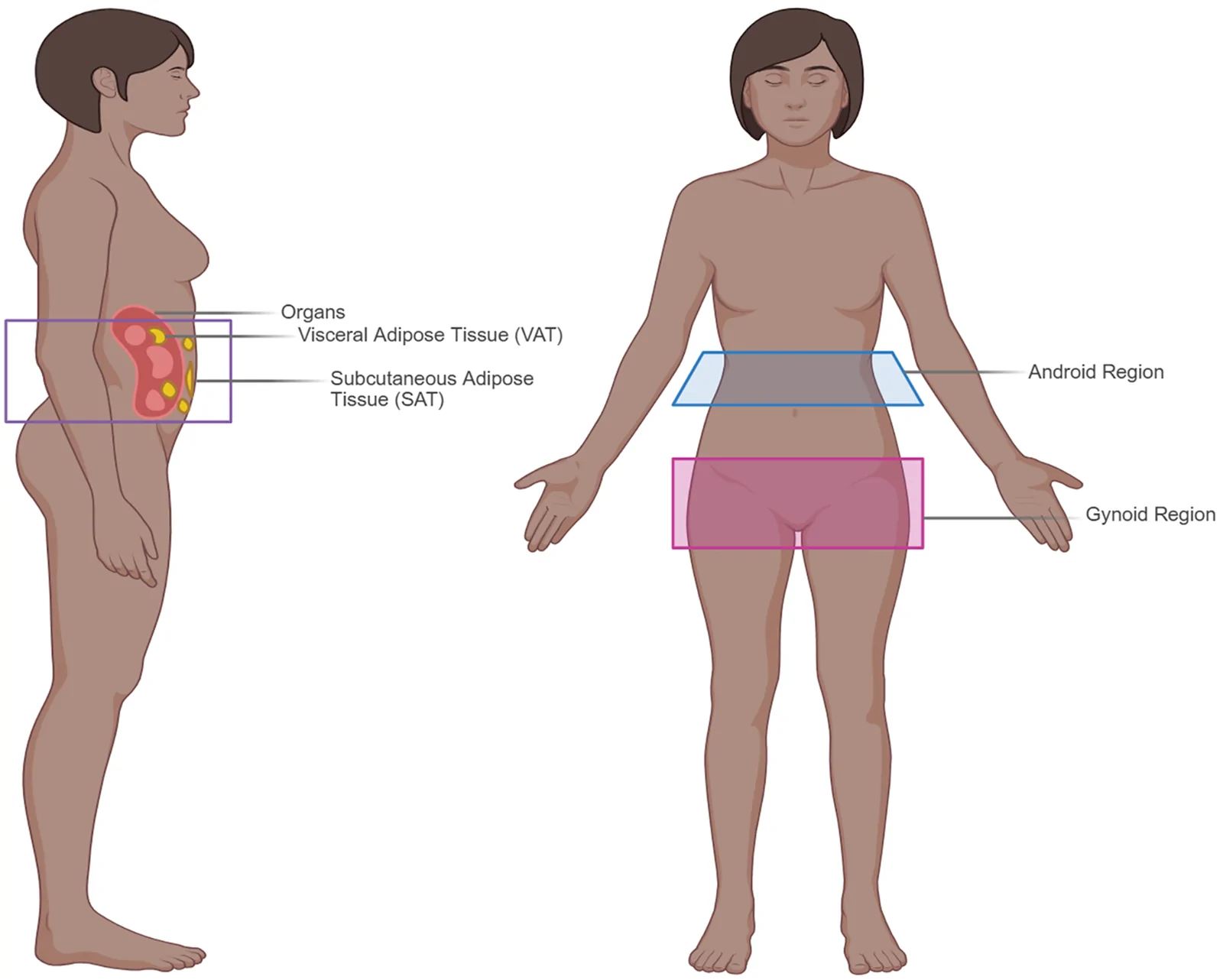
DXA-based body fat distribution metrics derived from GE Lunar Prodigy Primo DXA scanner (GE Healthcare, Wisconsin, USA). Illustration created in https://BioRender.com.

Whole-body DXA scans were obtained using a GE Lunar Prodigy Primo DXA scanner (GE Healthcare, Wisconsin, USA) according to the manufacturer’s guidelines (GE Healthcare 2017). Fig. 2 presents an approximation of each body fat distribution area for clarity.

We obtained algorithmic estimates of VAT and SAT mass (kg), volume (centimeters cubed [cm^3^]), and area (centimeters squared [cm^2^]) within the ‘android’ region of the body (i.e., region of the abdomen from the iliac crest to about 10 centimeters to its cephalad limit) (Kaul et al., 2012, Ergun et al., 2013).

Body fat distribution (i.e., body shape) was further obtained via DXA region of interest measures including android- and gynoid- % fat mass. Additionally, the android/gynoid (A/G) ratio comparing the % of adipose tissue around the android region to that around the gynoid region was computed by DXA software. An A/G ratio greater than one indicates greater fat distribution around the android region, and an A/G ratio less than one indicates greater fat distribution around the gynoid region.

To further characterize the morphology of the sample, we report DXA-based BMI (kg/m^2^), and body-tissue estimates including % fat mass, % lean soft tissue mass, and bone mineral content (kg).

#### 2.3.3. Performance-based and self-reported outcomes

Performance-based and self-reported outcomes were measured using reliable and valid performance-based assessments of mobility including walking initiation and upright mobility, walking speed, and walking endurance and self-reported assessments of anxiety and depressive symptoms, health-related quality of life, fatigue, and pain.

##### 2.3.3.1. Performance-based assessments.

Walking initiation and upright mobility were measured using the Timed Up and Go (TUG) (Nilsagard et al., 2007). Participants were instructed to stand from a chair without using their arms for support, walk around a cone, and then sit back down again, also without using their arms for support. They were asked to perform this task as quickly and safely as possible. Two trials were completed, and the average time in seconds was included in this manuscript. Shorter times indicate better performance. The TUG demonstrates excellent test-retest reliability and is widely used to assess balance and physical mobility in people with MS, capturing functional components such as walking initiation and upright movement transitions (Nilsagard et al., 2007).

Walking speed was assessed using the Timed 25-Foot Walk (T25FW) (Motl et al., 2017). Participants were instructed to walk as quickly and safely as possible across a marked 25-foot straight course from static start. Two trials were completed, and the average time in seconds was included in the manuscript. Shorter times indicate better performance. The T25FW demonstrates excellent test-retest reliability and strong validity as a measure of ambulatory function in people with MS (Motl et al., 2017).

Walking endurance was assessed using the Six-Minute Walk (6MW) (Goldman et al., 2008). Participants were asked to walk at their normal comfortable pace around a designated oval course free of obstacles for six minutes. A member of the research team followed behind the participant with a standard distance measuring wheel and subsequently recorded the total distance walked in meters. Longer distance indicates better performance. The 6MW demonstrates strong reliability and validity as a measure of walking endurance in people with MS (Goldman et al., 2008).

##### 2.3.3.2. Self-reported assessments.

Anxiety and depression were measured using the Hospital Anxiety and Depression Scale (HADS), a 14-item self-reported assessment divided into seven anxiety-related items and seven depression-related items (Honarmand and Feinstein, 2009). Items are rated using a four-point Likert-type scale (i.e., 0–4). After reverse scoring a subset of items, the items from the anxiety (HADS-A) and depression (HADS-D) sub-scales were summed independently, whereby higher scores indicated greater depression and anxiety symptoms in the past four weeks, respectively. The HADS demonstrates strong validity and good screening utility for detecting major depression and generalized anxiety disorder in people with MS (Honarmand and Feinstein, 2009).

Health-related quality of life (HRQOL) was measured using the Multiple Sclerosis Impact Scale (MSIS-29) (Riazi, 2002). The MSIS-29 is a 29-item self-reported assessment that measures MS-specific physical and psychological HRQOL. Items are rated using a five-point Likert-type scale (i.e., 1–5). The physical domain (MSIS Physical) was scored by summing scores for items 1–20, subtracting 20, dividing the difference by 80, and then multiplying by 100. The psychological domain (MSIS Psych) was scored by summing the scores for items 21–29, subtracting nine, dividing the difference by 36, and then multiplying by 100. Higher scores in both domains indicate a greater impact of MS on HRQOL in the past two weeks (i.e., worse HRQOL). The MSIS-29 demonstrates strong reliability and validity as a measure of MS-specific physical and psychological HRQOL (Riazi, 2002).

Perceived fatigue was measured using the Fatigue Severity Scale (FSS) (Learmonth et al., 2013). The FSS is a nine-item self-reported assessment rated using a 7-point Likert-type scale (i.e., 1–7). The items were averaged to generate a measure of fatigue severity that ranged between 1–7, whereby higher scores indicated greater fatigue severity within the past week. The FSS demonstrates acceptable reliability and strong validity as a measure of fatigue severity in people with MS (Learmonth et al., 2013).

Pain was measured using the short-form of the McGill Pain Questionnaire (SF-MPQ) (Strand et al., 2008). The SF-MPQ is a 15-item self-reported assessment that assesses sensory (e.g., ‘stabbing’, ‘sharp’) and affective (e.g., ‘sickening’, ‘tiring-exhausting’) dimensions of whole-body pain intensity. Items are rated using a 4-point Likert-type intensity scale (i.e., 0–3). The items were summed to produce an overall measure of pain intensity, with higher scores indicating greater pain intensity over the past week. The SF-MPQ demonstrates acceptable reliability and validity as a multidimensional measure of pain intensity and quality across clinical populations, like MS (Strand et al., 2008).

### 2.4. Data analysis

Data analyses were performed in IBM SPSS, version 30. We report descriptive data for the demographic, clinical, and morphological characteristics of the sample as frequencies and percentages for categorical variables n(%) and as mean with standard deviations for continuous variables *M(SD)*. We further provide descriptive data on DXA-based body fat distribution metrics and performance-based and self-reported outcomes as *M(SD)*. We report anthropometric and body composition data for the overall sample and sex-specific sub-groups.

To assess the associations among DXA-based body fat distribution metrics and performance-based and self-reported outcomes of MS, we estimated Spearman rank-order bivariate correlations among VAT and SAT mass (kg), volume (cm^3^), and area (cm^2^), android- and gynoid- % fat mass, and A/G ratio with TUG (average seconds), T25FW (average seconds), 6MW (distance walked in meters), HADS-A, HADS-D, MSIS Physical, MSIS Psych, FSS, and SF-MPQ. The Spearman correlation avoids effects of outliers and/or non-normal data on bivariate associations (Schober et al., 2018). We further performed partial Spearman correlations controlling for sex and age as possible confounding variables of body fat distribution (Karastergiou, 2019, Hunter et al., 2010). The partial Spearman correlations accounted for the joint influence of confounding variables on the association between paired variables in the analyses rather than only the outcome variable in regression analyses. Values for the correlation coefficients were interpreted as small (.1 – .29), moderate (.3 – .49), and large (.5+) (Cohen, 1988). Due to an inflated experiment-wide error rate from the number of correlations, statistical inference is interpreted cautiously in this report, as it should be verified through future confirmatory studies (Bender and Lange, 2001).

## 3. Results

Descriptive demographic, clinical, and morphological characteristics of the study sample are presented in Table 1 in full. The sample comprised 57 individuals with MS and was predominately female (70.2 %). The *M(SD)* age was 48.0(11.2) years and most participants identified as white/Caucasian (68.4 %). The *M(SD)* disease duration was 12.8 (7.3) years, and the duration of MS symptomology was 15.8(9.8) years. The majority of participants were diagnosed with relapsing-remitting MS (RRMS; 91.2 %). Overall, 70.2 % of participants were taking DMTs (21.0 % ocrelizumab [Ocrevus], 12.3 % dimethyl fumarate [Tecfidera], 7.0 % glatiramer acetate [Copaxone/Glatopa], 7.0 % fingolimod [Gilenya], 7.0 % natalizumab [Tysabri], 3.5 % teriflunomide [Aubagio], 3.5 % siponimod [Mayzent], 3.5 % interferon beta-1a [Rebif], 1.8 % interferon beta-1b [Betaseron], 1.8 % ofatumumab [Kesimpta], and 1.8 % cladribine [Mavenclad]). The *M(SD)* EDSS score was 2.9(1.6) (i.e., minimal to moderate disability). The DXA-based BMI for the overall sample was *M(SD)* 27.5(5.5) kg/m^2^, with values of 27.6(5.6) for women and 27.3(5.5) for men, indicating that most participants were in the ‘overweight’ category (Number of people with MS | Atlas of MS 2000). The *M(SD)* DXA-based % fat mass was 41.2(7.5) % for women and 28.2 (7.5) % for men, with both sexes meeting clinically relevant thresholds for classification as ‘overweight’ (Potter et al., 2025).

DXA-based body fat distribution metrics, including VAT and SAT mass, volume, and area, as well as android and gynoid % fat mass and the android/gynoid (A/G) ratio, are presented in Table 2, with values stratified by sex. Performance-based and self-reported outcomes included in the main analyses are also summarized in Table 2.

Spearman bivariate correlations and partial correlations among the DXA-based body fat distribution metrics and performance-based and self-reported outcomes are presented in Tables 3 and 4. There was generally a lack of statistically significant correlations, and the magnitude of correlations was generally small. Significant small to moderate correlations were observed between gynoid % fat mass and HADS-D (*r*_*s*_ = −.28, *p* = .033) and MSIS Physical (*r*_*s*_ = −.31, *p* = .018). However, gynoid % fat mass was no longer associated with HADS-D (*pr*_*s*_ = −.19, *p* = .163) or MSIS Physical (*pr*_*s*_ = −.22, *p* = .104) when controlling for covariates including sex and age. There were no other significant correlations observed between the DXA-based body fat distribution metrics and the performance-based and self-reported outcomes. We replicated those bivariate analyses controlling for EDSS, and there were still no associations between the DXA-based body fat distribution metrics and the outcomes. We further examined each outcome using multivariable linear regression models with and without sex, age, and EDSS as covariates, and none of the models were statistically significant; the results were consistent with the bivariate and partial Spearman correlations.

## 4. Discussion

This exploratory, cross-sectional, study examined the associations among DXA-based body fat distribution metrics and frequently studied outcomes of MS. Of the DXA-based body fat distribution metrics analyzed, only gynoid % fat mass was significantly associated with two of the outcomes, yielding inverse associations with depressive symptoms and physical health-related quality of life that were non-significant when controlling for sex and age. Overall, this indicated that none of the DXA-based body fat distribution metrics were associated with performance-based and self-reported outcomes of MS. Collectively, such null results for body fat distribution, combined with the null results of the larger body of research on *overall* body fat, suggests that body fatness as a metric of body composition may not represent a potent correlate of MS outcomes (Jeng and Motl, 2022, Silveira et al., 2020). This may indicate that symptom burden, disease heterogeneity, comorbidities, and other physiological factors (e.g., cardiorespiratory and muscular fitness) exert stronger influences on MS outcomes than body composition alone (Marrie et al., 2023, Jeng and Motl, 2022, Silveira et al., 2020, Heine et al., 2016, Du et al., 2024).

Our sample largely reflects the demographic and clinical profile of the MS population, with most participants identifying as female, white/Caucasian, middle-aged adults with RRMS, and minimal to moderate disability (based on EDSS scores) (Wallin et al., 2019, Kurtzke, 2015, Hittle et al., 2023, Langer-Gould et al., 2022, Portaccio et al., 2024). We further enrolled the sample based on BMI for yielding a broad range of scores from DXA-based body fat distribution, and this avoided a truncated distribution as a source of the null results. We compared body fat and its distribution from DXA in our sample with normative data for the general population, as this study did not include a non-MS control sample. Compared with white/Caucasian adults aged 40–49 years from the general United States population, % fat mass and % lean soft tissue mass in our sample were similar and corresponded with approximately the 50^th^ and 60^th^ percentiles for women and men, respectively (Imboden et al., 2017, Imboden et al., 2017). Among male participants, % fat mass was slightly lower (28.2 % vs. 30.1 %) and % lean soft tissue mass modestly higher (69.1 % vs. 67.5 %) than age-matched reference standards, whereas A/G ratios for both women and men exceeded those of the general population (.9 vs .4 and 1.1 vs .7, respectively) (Imboden et al., 2017, Imboden et al., 2017). This is partially consistent with prior research whereby people with MS have worse body composition across all three tissue compartments than non-MS controls (Pilutti et al., 2025). Nevertheless, it remains difficult to determine whether the body composition and fat distribution patterns in our sample are truly representative given the absence of DXA-based reference standards for the MS population.

The results from this study are not consistent with research in non-MS populations linking body fat distribution, particularly VAT, with physical and psychological health (Chua et al., 2021, Coín-Aragüez et al., 2018, Everson-Rose et al., 2009, Cosan et al., 2021). Such research has emphasized VAT’s pro-inflammatory effects, implicating it in outcomes such as anxiety and depression, conditions that are prevalent in MS and are associated with disease-specific outcomes (Marrie et al., 2023, Coín-Aragüez et al., 2018, Everson-Rose et al., 2009). Despite the immune-mediated pathophysiology of MS and VAT’s inflammatory characteristics, we observed no associations between VAT metrics, % android fat mass, A/G ratio and performance-based or self-reported outcomes in our sample. One possible explanation is that DMTs targeting inflammation in MS may attenuate or obscure VAT-related inflammatory effects that would otherwise influence MS outcomes (Robertson and Moreo, 2016, Zhang et al., 2021). It is further plausible that VAT-related inflammation does not substantially affect disease mechanisms (e.g., neurodegeneration) in ways that translate to measurable symptoms of MS, even though structural associations are observed in non-MS populations. For example, a study of 10,001 non-MS individuals reported that higher VAT and SAT predicted lower brain volumes on magnetic resonance imaging (MRI) (GE Healthcare 2023). Another consideration is that mobility-related outcomes may be more sensitive to adiposity among individuals with severe obesity (e.g., BMI > 35) (Vincent et al., 2010). Given our sample had a mean(SD) BMI of 27.5 (5.5) and a range of 18.3–42.1, the distribution may have limited our ability to detect such effects.

Overall, our results support the broader literature suggesting that body composition may play a more modest and context-dependent influence on MS outcomes (Kidwell-Chandler et al., 2025). Notably, body composition and body fat distribution intrinsically differ between the sexes and over the lifespan (Karastergiou, 2019, Imboden et al., 2017, Imboden et al., 2017). Higher BMI in early life is associated with MS risk, with a potentially stronger association among women (Gianfrancesco et al., 2017). In contrast, men with MS often experience more rapid disability progression, poorer outcomes, and greater body fat accumulation, trends that may compound with aging (Pilutti et al., 2025, Simmons et al., 2021). The multifactorial contributions of sex, age, and obesity-related comorbidities underscore the importance of further examining the relationship between body composition and MS outcomes and disease progression. Additionally, this highlights the importance of applying gold-standard measures of body composition, such as DXA, whenever practicable, given the limitations of BMI in MS. Indeed, BMI becomes less reliable in older adults, does not adequately account for sex-based body composition differences, and notably underestimates adiposity in MS (Pilutti and Motl, 2016, Jabre and Bland, 2021). Future research should therefore examine body composition using longitudinal and sex-stratified designs, and perhaps consider incorporating upstream biomarkers, such as brain structure and function measured by MRI or relevant blood-based inflammatory markers for further mechanistic insight.

### 4.1. Limitations

There are several limitations to the present study. This study was a priori designed based on the relationship between oxygen cost of walking and body composition in MS (Jeng et al., 2023). As such, the eligibility criteria and original sample size were catered to the needs of the original study. Future research applying more sensitive study designs and greater statistical power may better delineate if body fat distribution relates with frequently studied outcomes of MS. We further note that eligibility based on inclusion/exclusion criteria was confirmed through a self-report screening questionnaire, and the criteria including diagnosis of MS, were self-reported and were not verified by the research team. The cross-sectional design of this study limits causal inferences and constrains the ability to assess sex- and age-related differences over time. We did not assess blood-based inflammatory markers linked with adipose tissue and MS, and future studies may benefit from incorporating such markers when examining body fat and its distribution in MS. This study did not report specific data on comorbidities or use of other medications beyond DMTs, because these variables were not collected. Obesity is a relevant risk factor for comorbidities, and comorbidities influence MS outcomes and progression. Future studies should more thoroughly disentangle intricate associations among body fatness, comorbidities, and outcomes in MS. Additionally, it is possible that DMTs targeting immune cells responsible for inflammation may mute the effects of VAT-related inflammation. To date, the impact of DMTs on VAT-related inflammation has not been studied; as such, future research may elucidate this potential confounder.

## 5. Conclusion

Although body fat distribution, particularly VAT, represents a biologically plausible and clinically relevant characteristic in the context of MS, our results did not support its association with MS outcomes. When controlling for sex and age, no significant relationships were observed between any of the DXA-based body fat distribution metrics and performance-based and self-reported outcomes of MS, including mobility, anxiety and depressive symptoms, health-related quality of life, fatigue, and pain in the present study. This study represents an initial exploratory analysis of DXA-based body fat distribution and frequently studied MS outcomes. Future confirmatory research should contextualize adiposity in MS by examining possible associations, including those with preclinical biomarkers, sex-stratified aging trajectories, and comorbidities, and evaluating DMTs as potential confounders. Such research might better inform the role of body composition, notably body fat and its distribution, in MS.

## Figures and Tables

**Table 1:** Demographic, clinical, and morphological characteristics among 57 people with multiple sclerosis.

Variable		*n (%)*	*M(SD), Min-Max*
Sex	Female	40(70.2)	–
	Male	17(29.8)	
Age (yrs)	Overall	–	48.0(11.2), 24.0–74.0
	Female	–	47.4(11.2), 24.0–74.0
	Male	–	48.0(11.6), 27.0–68.0
Race	White/Caucasian	39(68.4)	^−^
	Black/African American	16(28.1)	
	Other	2(3.5)	
Disease duration (yrs)		–	12.8(7.3), 1.0–35.0
Duration of symptoms (yrs)		–	15.8(9.8), 1.0–46.0
MS Type	Relapsing-remitting	52(91.2)	–
	Secondary Progressive	2(3.5)	
	Primary Progressive	2(3.5)	
	Benign	1(1.8)	
Disease Modifying Therapy	None	17(29.8)	–
	ocrelizumab (Ocrevus)	12(21.0)	
	dimethyl fumarate (Tecfidera)	7(12.3)	
	glatiramer acetate (Copaxone/Glatopa)	4(7.0)	
	fingolimod (Gilenya)	4(7.0)	
	natalizumab (Tysabri)	4(7.0)	
	teriflunomide (Aubagio)	2(3.5)	
	siponimod (Mayzent)	2(3.5)	
Variable		*n (%)*	*M(SD), Min-Max*
Disease Modifying Therapy *(continued)*	interferon beta-1a (Rebif)	2(3.5)	–
	interferon beta-1b (Betaseron)	1(1.8)	
	ofatumumab (Kesimpta)	1(1.8)	
	cladribine (Mavenclad)	1(1.8)	
EDSS (0–10)	Overall	–	2.9(1.6), .0–6.5
	Female		2.8(1.6), .0–6.5
	Male		3.2(1.7), .0–6.5
BMI (kg/m^2^)	Overall	–	27.5(5.5), 18.3–42.1
	Female		27.6(5.6), 19.0–42.1
	Male		27.3(5.5), 18.3–40.3
% Fat mass	Overall	–	37.3(9.5), 9.64–54.0
	Female		41.2(7.5), 23.2–54.2
	Male		28.2(7.5), 9.64–39.41
% Lean soft tissue mass	Overall	–	60.6(8.9), 44.6–82.2
	Female		57.0(7.0), 44.6–73.5
	Male		69.1(6.9), 58.0–82.2
Bone mineral content (kg)	Overall	–	2.5(.4), 1.5–3.6
	Female		2.3(.4), 1.5–3.1
	Male		2.9(.3), 2.4–3.6

*Note:* MS = multiple sclerosis; EDSS = Expanded Disability Status Scale; BMI = body mass index; yrs = years; kg = kilograms; m^2^ = meters squared. Values are shown as frequency and percent *n(%)*, mean (*M*), standard deviation (*SD*), minimum (*Min*), and maximum (*Max*) respectively.

**Table 2:** Dual-energy X-ray absorptiometry (DXA)- based body fat distribution metrics and performance-based and self-reported outcomes among 57 people with multiple sclerosis.

Variable		*M(SD), Min-Max*
*DXA-based Body Fat Distribution Metrics*		
VAT mass (kg)	Overall	.7(.6), .0–2.2
	Female	.7(.6), .0–2.2
	Male	.9(.6), .0–1.9
VAT volume (cm^3^)	Overall	794.1(620.1), 14.0–2371.0
	Female	747.4(608.2), 14.0–2371.0
	Male	903.9(652.7), 31.0–2048.0
VAT area (cm^2^)	Overall	87.7(66.2), 2.0–246.0
	Female	84.0(65.2), 2.0–246.0
	Male	96.5(69.7), 4.0–214.0
SAT mass (kg)	Overall	1.8(.9), .1–4.2
	Female	1.9(.9), .5–4.2
	Male	1.5(1.0), .1–3.7
SAT volume (cm^3^)	Overall	1894.3(998.2), 140.0–4419.0
	Female	2023.5(970.7), 499.0–4419.0
	Male	1590.6(1025.0), 140.0–3932.0
SAT area (cm^2^)	Overall	211.1(113.1), 15.0–552.0
	Female	229.0(111.1), 54.0–552.0
	Male	169.1(109.7), 15.0–424.0
Android % fat mass	Overall	39.0(12.1), 6.9–62.0
	Female	41.8(11.2), 17.6–62.0
	Male	32.3(11.9), 6.9–50.8
Variable		*M(SD), Min-Max*
Gynoid % fat mass	Overall	40.2(10.7), 13.8–56.6
	Female	45.3(7.1), 28.7–56.6
	Male	28.1(7.2), 13.8–39.0
A/G ratio	Overall	1.0(.2), .4–1.5
	Female	.9(.2), .5–1.1
	Male	1.1(.3), .4–1.5
*Performance-based Outcomes*		
TUG (s)		7.7(4.5), 4.5–32.9
T25FW (s)		5.2(3.2), 3.3–25.0
6MW (m)		392.7(92.7), 91.8–560.5
*Self-reported Outcomes*		
HADS-A		5.8(3.9), .0–17.0
HADS-D		4.5(4.0), .0–15.0
MSIS Physical		20.4(19.5), .0–68.75
MSIS Psych		22.3(19.8), .0–80.56
FSS		3.9(1.6), 1.0–6.9
SF-MPQ		5.3(6.1), 0–30.0

*Note*: VAT = visceral adipose tissue; SAT = subcutaneous adipose tissue; A/G = android/gynoid; TUG = Timed Up and Go; T25FW = Timed 25-Foot Walk; 6MW = Six-Minute Walk; HADS-A = Hospital Anxiety and Depression Scale – Anxiety sub-scale; HADS-D = Hospital Anxiety and Depression Scale – Depression sub-scale; MSIS Physical = Multiple Sclerosis Impact Scale – Physical sub-scale; MSIS Psych = Multiple Sclerosis Impact Scale – Psychological sub-scale; FSS = Fatigue Severity Scale; SF-MPQ = short form of the McGill Pain Questionnaire; m = meters; kg = kilograms; cm = centimeters; % = percent; s = seconds. Values are shown as mean (*M*), standard deviation (*SD*), minimum (*Min*) and maximum (*Max*) respectively.

**Table 3:** Spearman’s rank-order correlations and partial correlations between dual-energy X-ray absorptiometry (DXA)- based VAT and SAT and performance-based and self-reported outcomes among 57 people with multiple sclerosis.

		VAT mass (kg)		VAT volume (cm^3^)		VAT area (cm^2^)		SAT mass (kg)		SAT volume (cm^3^)		SAT area (cm^2^)	
		*r*_*s*_	*pr*_*s*_	*r*_*s*_	*pr*_*s*_	*r*_*s*_	*pr*_*s*_	*r*_*s*_	*pr*_*s*_	*r*_*s*_	*pr*_*s*_	*r*_*s*_	*pr*_*s*_
*Performance-based Outcomes*													
TUG (s)		.00	−.08	.00	−.08	−.02	−.09	−.04	.01	−.04	.01	−.02	.03
	*p*	.994	.578	.989	.563	.898	.502	.795	.944	.758	.948	.871	.820
T25FW (s)		−.03	−.09	−.03	−.09	−.04	−.10	−.01	.01	−.03	.00	.01	.04
	*p*	.847	.520	.837	.510	.785	.468	.946	.922	.849	.996	.920	.781
6MW (m)		.04	.12	.04	.12	.04	.12	.18	.15	.20	.17	.16	.13
	*p*	.790	.401	.771	.385	.753	.382	.173	.260	.128	.211	.234	.350
*Self-reported Outcomes*													
HADS-A		−.15	−.08	−.15	−.08	−.15	−.08	−.02	−.09	.01	−.08	.02	−.08
	*p*	.257	.578	.260	.581	.262	.544	.910	.508	.972	.571	.909	.583
HADS-D		−.02	−.08	−.02	−.08	−.04	−.10	−.24	−.20	−.25	−.20	−.21	−.16
	*p*	.903	.577	.902	.579	.762	.487	.071	.144	.062	.135	.111	.244
MSIS Physical		−.06	−.12	−.06	−.12	−.08	−.13	−.18	−.13	−.18	−.13	−.21	−.15
	*p*	.666	.396	.654	.388	.553	.336	.190	.337	.179	.335	.126	.273
		VAT mass (kg)		VAT volume (cm^3^)		VAT area (cm^2^)		SAT mass (kg)		SAT volume (cm^3^)		SAT area (cm^2^)	
		*r*_*s*_	*pr*_*s*_	*r*_*s*_	*pr*_*s*_	*r*_*s*_	*pr*_*s*_	*r*_*s*_	*pr*_*s*_	*r*_*s*_	*pr*_*s*_	*r*_*s*_	*pr*_*s*_
MSIS Psych		−.04	.01	−.04	.01	−.05	−.01	.01	−.01	.01	−.01	−.01	−.03
	*p*	.795	.943	.778	.961	.707	.961	.970	.934	.923	.960	.946	.847
FSS	*p*	−.06	−.04	−.06	−.03	−.07	−.05	−.04	−.05	−.02	−.04	−.04	−.06
		.653	.801	.660	.808	.602	.738	.797	.711	.873	.772	.752	.654
SF-MPQ		.05	.10	.04	.10	.03	.09	.11	.09	.10	.08	.09	.06
	*p*	.735	.450	.749	.462	.805	.520	.413	.528	.442	.580	.515	.658

*Note*: VAT = visceral adipose tissue; SAT = subcutaneous adipose tissue; TUG = Timed Up and Go; T25FW = Timed 25-Foot Walk; 6MW = Six-Minute Walk; HADS-A = Hospital Anxiety and Depression Scale – Anxiety sub-scale; HADS-D = Hospital Anxiety and Depression Scale – Depression sub-scale; MSIS Physical = Multiple Sclerosis Impact Scale – Physical sub-scale; MSIS Psych = Multiple Sclerosis Impact Scale – Psychological sub-scale; FSS = Fatigue Severity Scale; SF-MPQ = short form of the McGill Pain Questionnaire; m = meters; kg = kilograms; cm = centimeters; s = seconds. Partial correlations control for age and sex.

* *p* < .05;

** *p* < .01

**Table 4:** Spearman’s rank-order correlations and partial correlations between dual-energy X-ray absorptiometry (DXA)-based android % fat mass, gynoid % fat mass, A/G ratio, disability, and performance-based and self-reported outcomes among 57 people with multiple sclerosis.

		Android % fat mass		Gynoid % fat mass		A/G ratio	
		*r*_*s*_	*pr*_*s*_	*r*_*s*_	*pr*_*s*_	*r*_*s*_	*pr*_*s*_
*Performance-based Outcomes*							
TUG (s)		−.04	.00	−.06	.03	.14	.07
	*p*	.765	.978	.644	.810	.296	.604
T25FW (s)		.04	.04	.01	.02	.09	.07
	*p*	.787	.787	.951	.871	.529	.636
6MW (m)		.05	.04	.10	.07	−.08	−.03
	*p*	.715	.796	.471	.599	.533	.818
*Self-reported Outcomes*							
HADS-A		−.01	−.11	.14	−.06	−.24	−.12
	*p*	.949	.443	.297	.662	.074	.388
HADS-D		−.17	−.11	−.28*	−.19	.14	.04
	*p*	.199	.428	.033	.163	.288	.772
MSIS Physical		−.18	−.11	−.31*	−.22	.13	.02
	*p*	.181	.416	.018	.104	.352	.901
MSIS Psych		−.03	−.03	−.09	−.13	.06	.09
	*p*	.829	.850	.507	.355	.683	.536
FSS		−.02	−.03	−.14	−.22	.10	.15
	*p*	.907	.856	.312	.105	.441	.288
SF-MPQ		.08	.07	.01	−.04	.01	.16
	*p*	.545	.598	.942	.768	.462	.231

*Note*: A/G = android/gynoid; TUG = Timed Up and Go; T25FW = Timed 25-Foot Walk; 6MW = Six-Minute Walk; HADS-A = Hospital Anxiety and Depression Scale – Anxiety sub-scale; HADS-D = Hospital Anxiety and Depression Scale – Depression sub-scale; MSIS Physical = Multiple Sclerosis Impact Scale – Physical sub-scale; MSIS Psych = Multiple Sclerosis Impact Scale – Psychological sub-scale; FSS = Fatigue Severity Scale; SF-MPQ = short form of the McGill Pain Questionnaire; m = meters; kg = kilograms; cm = centimeters; % = percent; s = seconds. Partial correlations control for age and sex.

* *p* < .05;

** *p* < .01

## Data Availability

The data included in the current manuscript are available from the corresponding author upon request.
